# Neighborhood Tax Foreclosures, Educational Attainment, and Preterm Birth among Urban African American Women

**DOI:** 10.3390/ijerph16060904

**Published:** 2019-03-13

**Authors:** Shawnita Sealy-Jefferson, Dawn P. Misra

**Affiliations:** 1 College of Public Health, Division of Epidemiology, The Ohio State University, Columbus, OH 43210, USA; 2 Department of Family Medicine and Public Health Sciences, Wayne State University School of Medicine, Detroit, MI 48202, USA; dmisra@med.wayne.edu

**Keywords:** preterm birth, neighborhood effects, tax foreclosures, urban decline, African American women, segregation, educational attainment

## Abstract

Ecological evidence suggests that neighborhoods with more tax foreclosures also have more adverse birth outcomes. However, whether neighborhood-level tax foreclosures impact individual-level risk for adverse birth outcomes is unknown. We assessed whether living in a neighborhood with high tax foreclosures is associated with a woman’s preterm birth (PTB) risk and tested for effect modification by educational attainment, among urban African American women from the Life Influence on Fetal Environments Study (2009–2011; *n* = 686). We linked survey and medical record data to archival, block-group level tax foreclosure data from the county treasurer. We used Modified Poisson regression with robust error variance and included a foreclosure X education interaction in adjusted models. In the overall sample, neighborhood tax foreclosures did not predict PTB (adjusted relative risk: 0.93, CI: 0.74, 1.16), but the association was modified by educational attainment (interaction *p* = 0.01). Among women with lower education (*n* = 227), neighborhood tax foreclosures did not predict PTB risk. The association for women with higher education (*n* = 401) was statistically significant for a reduction in risk for PTB (adjusted relative risk: 0.74, CI: 0.55, 0.98) among those who lived in neighborhoods with high versus low tax foreclosures. Future studies should seek to identify the mechanisms of this association.

## 1. Introduction

Preterm birth (PTB), or birth before 37 completed weeks of gestation, is the leading cause of infant mortality and is a significant cause of maternal and pediatric morbidity [1]. We have also seen troubling increases in overall PTB rates in recent years [2]. Persistent racial disparities in PTB exist, with African American women being more than twice as likely as white women to have this adverse birth outcome [3,4]. Importantly, traditional risk factors for PTB (including behavioral and biologic factors) do not explain the disproportionate burden among African Americans [5]. Social exposures, arising from segregated residential environments, may explain the longstanding racial disparities in PTB in the U.S. [6].

Urban decline (or shrinkage) is commonly associated with postindustrial Metropolitan areas in the U.S. and Europe [7]. Urban decline has been associated with high crime rates, poverty, and deteriorated built environments [8]. Since 1958, Detroit, MI has experienced a nearly 79% decline in property values and is a particularly revealing example of urban decline [9,10]. The economic decline of the late 20th century has plagued deindustrialized cities like Detroit, Philadelphia, and Baltimore, resulting in chronic housing abandonment [11]. Research on the impact of urban decline in Detroit may provide clues about what might be happening, but may be more difficult to empirically document, in other areas of the United States [12]. 

Tax delinquency is a common indicator of housing abandonment [11,13] and signifies a level of neighborhood disinvestment in which the homeowner either believes further investment in the home is useless, or lacks the financial resources to improve the home [14]. Residents of urban areas plagued by concentrated poverty and disadvantage often experience neighborhood distress from properties in which homeowners have ceased carrying out at least one major property ownership responsibility, and as a result, the properties are vacant or prone to imminent vacancy [14]. Americans have not experienced this magnitude of property tax foreclosures since the Great Depression [15].

To our knowledge, one existing study investigated the impact of neighborhood tax delinquency on adverse birth outcomes, using administrative data for both neighborhood distress measures and adverse birth outcomes. The researchers found that neighborhood-level housing abandonment measures (specifically tax delinquency) explained more variation in neighborhood-level adverse birth outcomes than other neighborhood-level census-based socioeconomic disadvantage measures (such as percent poverty and unemployment) [16]. Furthermore, they reported that tax delinquency was a significant predictor of three adverse birth outcomes (including PTB, low birthweight, and infant mortality), and associations were independent of other measures of neighborhood distress [16]. Future research on this topic, using individual-level data, will increase our understanding of the ways in which living in a neighborhood with blight and disinvestment impacts risk of PTB and allow us to disentangle neighborhood and individual effects. 

Social determinants of perinatal health are complex and often interact to affect outcomes [17]. In particular, educational attainment is the most basic socioeconomic measure, given that it shapes occupational opportunity and earning potential and is protective against adverse health outcomes [18]. We previously reported evidence of joint effects between subjective neighborhood measures (including social disorder, food availability, walkability, and social cohesion) and educational attainment on PTB risk among African American women [19]. Specifically, we found that women’s perceptions of their neighborhood context significantly predicted PTB among women with lower educational attainment, but not women with higher levels of education [19]. Women with less education may be more susceptible to the stress caused by living in neighborhoods they perceive as disadvantaged, whereas women with higher levels of education may have resources to buffer against neighborhood stressors.

To extend the literature on neighborhood tax foreclosures and adverse birth outcomes, we examined the independent impact of neighborhood tax foreclosures on risk of PTB among African American women from the Life Influences on Fetal Environments Study (LIFE). We also examined whether educational attainment buffered against the impact of neighborhood tax foreclosures on risk of PTB. We hypothesized that neighborhood tax foreclosures would have a joint effect with educational attainment to predict risk of PTB among African American women, with higher educational attainment acting as a buffer. 

## 2. Materials and Methods

### 2.1. Sample

The original LIFE study was a retrospective cohort of self-identified African American women (18+ years old) from Metropolitan Detroit, Michigan (2009–2011) [19]. The primary objective of the LIFE study was to examine whether and how racism is associated with PTB. Exclusion criteria included: (1) Non-English speaker, (2) intellectual disabilities, serious cognitive deficits, or evidence of mental illness, based on history or any prior records. Women were interviewed in-person during their postpartum hospital stay, and their medical history was abstracted from medical records. The final sample of the original LIFE study included 1410 women, which represented 71% of the women approached for study participation. The analytic sample for current cross-sectional analysis was restricted to women who resided in the city of Detroit (*n* = 686, 48% of the original sample). The LIFE study was approved by the institutional review boards at the University of Michigan (HUM00020527), St. John Providence Health Systems (173317-4), and Wayne State University (104908B3F). All study participants gave written informed consent.

### 2.2. Outcome Ascertainment

PTB was defined as birth before 37 completed weeks of gestation. We used a hierarchical algorithm to categorize gestational age, obtained from the medical records, and gave priority to the estimate based on early ultrasounds (between 6 and 20 weeks of gestation), as this is considered the most valid measure [20,21].

### 2.3. Exposure Ascertainment

An absolute number of adverse built environment features (e.g., grocery stores, fast food restaurants) has been linked to health outcomes [22,23,24]. Given the rapid neighborhood deterioration following the last recession, as well as the stark decline in population size in Detroit, population average estimates of home occupancy, number of residential parcels, or population counts from administrative sources would likely provide an inaccurate denominator for tax foreclosure rate calculations. Given this, we geocoded the current addresses of study participants using ArcGIS and matched and spatially linked the exact latitude and longitude of each address to archival tax foreclosure data from the Wayne County Treasurer (ascertained from Data Driven Detroit). We linked the absolute density (number) of tax foreclosures per block group to the LIFE dataset, based on the year study participants enrolled in the study (for instance, tax foreclosures occurring in 2009 were linked to LIFE study participants who enrolled in 2009).

#### Subjective and Objective Neighborhood Measures

Study participants reported detailed characteristics of their current neighborhood, using valid and reliable multi-item scales [19]. We examined five scale variables (Table 1) which measured subjective neighborhood social cohesion and trust, healthy food availability, walking environment, social disorder, and danger. We previously created a neighborhood disadvantage index (NDI), using principal components analysis of 9 optimally weighted 5-year block-group level estimates of variables from the American Community Survey (2007–2011), to examine objective neighborhood context (including racial and economic segregation) for the LIFE study participants [25]. The NDI included %: below poverty, unemployed, receiving public assistance, African American, female-headed households, college-educated, owner-occupied homes, as well as median income and home values [25]. A higher score on the NDI represented more disadvantage. Factor loadings were the highest for median income (84%) and lowest for % of owner-occupied homes (42%).

### 2.4. Effect Modifier

Our educational attainment variable included the highest level of education reported by study participants.

#### Covariates

Where people live is a nonrandom process, and residential selection is predicted by individual characteristics like age, race/ethnicity, and socioeconomic status [26,27]. In our prior work using data from the LIFE study, we identified very few associations between traditional risk factors and PTB in this study population [19,28], and income was the only variable we could consider a true confounder. As a result, we included the following predictors of residential selection in adjusted models: Age (<35 and ≥35 years), household income (<$35,000, ≥$35,000/year), and marital status (married, unmarried). We also controlled for length of residence in the current neighborhood (<24 and ≥24 months). 

### 2.5. Statistical Analysis

We used univariate and bivariate statistics for data description and used chi-square and Wilcoxon rank sum tests to quantify differences in categorical and continuous variables, respectively. We based cut-points on the distributions in the analysis sub-sample. The relationship between all neighborhood context measures was quantified with Spearman correlations. We assessed all variables for missing data (missing ranged from 0–11%), and list-wise deletion was performed. Since the prevalence of our outcome was >10%, and there was small block-group level variation in PTB which would preclude hierarchical modeling (ICC = 5.7%) [29], we used modified Poisson regression models with robust error variance [30,31] and estimated unadjusted and adjusted relative risks (RR) and associated 95% confidence intervals (95% CI) for the association between neighborhood tax foreclosures and risk of PTB. The neighborhood tax foreclosure variable was modeled continuously and was rescaled by the interquartile range, to allow us to interpret the results as the risk of PTB in women who resided in neighborhoods with high (75th percentile of the distribution) versus low (25th percentile of the distribution) tax foreclosures. We included an interaction term in adjusted models to test heterogeneity of the association by educational attainment, and we present stratum-specific results if warranted. Non-positivity occurs when certain segments of a study population only experience one level of the exposure [32,33,34]. We examined the tabular distributions of quintiles of the neighborhood tax foreclosure variable by educational attainment and confirmed that women with ≤12 and >12 years of education had a positive probability of residing in neighborhoods across the entire tax foreclosure spectrum [35]. We used the SAS version 9.4 for Windows (SAS Institute Inc., Cary, NC, USA) for all analyses. Two-sided *p*-values <0.05 and 95% confidence intervals that excluded 1 were considered statistically significant.

## 3. Results

The mean age of the study participants was 27 years old, and 16.5% had a PTB (*n* = 113) (which was the same proportion of preterm births to African American women in the United States in 2011) (Table 2). Nearly 80% of the sample were unmarried, and two thirds had at least 12 years of education. Over half of the sample had an annual income <$35,000/year and lived in their current neighborhood for 2+ years. None of the demographic variables examined were statistically significant predictors of PTB, in bivariate models. We observed no statistically significant bivariate associations between risk of PTB and either our objective NDI or the five subjective neighborhood measures.

Study participants resided in 383 block groups, with 1 to 7 women per block group. There were between 0 and 69 tax foreclosed properties per block group (median: 8). A total of 12.8% of block groups experienced no tax foreclosures, and neighborhoods categorized as having “high tax foreclosures” ranged from 13–69 per block group (data not shown). We observed weak correlations between neighborhood tax foreclosures and our subjective and objective neighborhood measures. The smallest correlation with tax foreclosures was with % of African Americans per block group: 0.09, and the largest was with % of college graduates per block group: −0.33 (data not shown).

In the overall sample, we observed no statistically significant association between the number of tax foreclosures and risk of PTB (adjusted RR: 0.93, 95% CI: 0.74, 1.16); however, there was evidence of effect modification by education attainment (*p* for interaction: 0.01) (Table 3). Though not significant, women who had ≤12 years of education, and resided in neighborhoods with high tax foreclosures had a higher risk of PTB than women with ≤12 years of education who lived in neighborhoods with low tax foreclosures (adjusted RR: 1.31, 95% CI: 0.95, 1.82). For women with >12 years of education, those who lived in neighborhoods with high tax foreclosures had significantly lower PTB risk than their counterparts who lived in neighborhoods with low tax foreclosures (adjusted RR: 0.74, 95% CI: 0.55, 0.98). In sensitivity analyses which excluded the *n* = 25 women who themselves experienced a foreclosure, the results were not appreciably different from the main analyses.

## 4. Discussion

The main finding from this study was that there was no independent statistically significant association between the number of neighborhood tax foreclosures and risk of PTB among urban African American women, but the association was significantly modified by educational attainment. Specifically, among women with >12 years of education, those who lived in neighborhoods with high tax foreclosures had a nearly 25% lower risk of PTB than women who lived in neighborhoods with low tax foreclosures, after accounting for predictors of residential selection and time lived in the current neighborhood. Among women with ≤12 years of education, though not statistically significant, a suggestive positive association between tax foreclosures and PTB risk was observed. We also found weak correlations between the number of tax foreclosures and several subjective and objective neighborhood measures. The number of tax foreclosures was least correlated with the % of African Americans in the neighborhood and most correlated with the % of college graduates.

The one existing study of neighborhood tax delinquency and neighborhood level adverse birth outcomes found significant preliminary evidence of an association [16]. Their independent variable was defined as “a percentage of parcels in a neighborhood that are delinquent on city and school district taxes averaged between the years 2004 and 2010, with the rate calculated as a percentage of taxable parcels- including all land use types” [16]. Our work extends this literature because we used individual-level birth outcome data, tested for effect heterogeneity by a health promoting social factor, quantified the absolute impact of protracted urban decline on risk of PTB, and used data from an entirely African American cohort. Within-group analyses, like ours, are uniquely equipped to identify risk as well as protective factors for adverse health outcomes among high risk groups and may inform etiologic research. The results of this research should be useful for hypothesis generation and will inform future research on this topic.

An increasing body of literature suggests that residential segregation is a fundamental (or root) cause of racial disparities in health [36,37,38,39], because African Americans are more likely to reside in disadvantaged neighborhoods (with concentrated poverty, disinvestment of resources, and infrastructure decay) than whites [37,40]. Residential segregation has been most persistent in regions with a high proportion of minority residents, with Black–white segregation in cities such as Detroit, Milwaukee, and New York remaining essentially unchanged over time [41,42]. In other words, the usual experience for these minority groups is to reside in highly segregated residential areas [43]. 

Contextually, the foreclosure crisis altered the social fabric of many neighborhoods in the U.S. [44,45,46,47,48,49], in ways that may be connected to stress and PTB risk. Research suggests that living in neighborhoods with high foreclosures is associated with poor physical and mental health [50,51,52,53]. Neighborhoods experiencing elevated home foreclosure also have high community stressors [54], like abandoned properties [55], crime [56], and decreased community resources, including home values and family wealth [55,57], tax revenue [54], stability [58], and social capital [59]. The “broken windows” theory of urban decline suggests that neighborhood physical disorder causes urban decay and serious crime, and is predictive of poor mental and physical health [60]. Neighborhoods plagued by this physical disorder may cause chronic stress among residents [55,57]. 

The way that homes come to be tax foreclosed in Detroit is worth discussing. First, the Delinquent Property Tax Foreclosure Public Act of 1999 mandates that properties are forfeited to the Wayne County Treasurer in their second year of tax delinquency; if the taxes remain unpaid, the foreclosure process commences on March 31st of the third year of delinquency [61]. Mortgaged properties are rarely foreclosed due to delinquent property taxes, because property taxes are usually included in monthly mortgage payments [62]. Furthermore, property tax foreclosures typically affect low income populations, including the elderly and individuals who inherit a property [62]. More than half of our study participants reported living in their current neighborhood for more than two years. Our neighborhood tax foreclosure measure captures extended disinvestment and neighborhood decline, which is likely stress-inducing for residents. Researchers have shown that from 2009 to 2015, the City of Detroit systematically violated the Michigan Constitution, by methodically inflating property tax assessments above their market values, affecting between 55% and 85% of properties in a given year [63]. They estimated that 10% of all tax foreclosures in Detroit from 2009 to 2015 were caused by unconstitutionally high property value assessments. More troublesome is that 25% of all tax foreclosures of the lowest valued homes were due to unconstitutional assessments [63]. Programs and policies that prevent owner-occupied homes from experiencing tax foreclosure will safeguard home ownership for low-income residents [62] and may decrease the spill-over effects on health for residents who live in neighborhoods plagued by housing abandonment.

Institutional racism occurs when the laws, policies, or customs of society or a group of institutions intentionally or inadvertently cause race-based inequities. African American homeowners, largely those with the lowest socioeconomic status, appear to be disproportionately impacted by illegally inflated property tax assessments, many of which result in tax foreclosure [63]. Contemporary discrimination is often hidden in plain sight, because it is embedded in social structures and institutions [64,65,66], and within policies that seem to be economic in nature and devoid of consequences for population health. 

In the current study, we found that high educational attainment appeared to buffer the effects of living in a neighborhood with high tax foreclosures on risk of PTB. A similar protective effect was not observed among women with lower levels of education (this sub-group had a suggestive increase in PTB risk associated with high neighborhood tax foreclosures). Educational attainment is the most basic socioeconomic measure, because it shapes future job prospects and earning potential [18]. Research has also shown that higher educational attainment is associated with more compliance with health recommendations during pregnancy [67,68]. Our results suggest that policies and programs which increase educational attainment among African American women may help buffer against the risk of PTB associated with living in a neighborhood with high tax foreclosures. Educational institutions may preserve societal power structures which establish social norms [69], but these institutions are also important determinants of health. Specifically, the pathways through which education may impact health may include neural development [70], biological aging [71,72,73], health behaviors and health literacy [74], sense of autonomy [75], as well as life chances (including through income and occupation). It is possible that women with higher education live in neighborhoods with high tax foreclosures, but the neighborhoods themselves are going through neighborhood development and revitalization. Future studies should seek to identify the mechanisms of this association. 

Importantly, both educational attainment and neighborhood tax foreclosures are social determinants of health that have clear policy relevance. The pathways through which high educational attainment and living in a neighborhood with high tax foreclosures jointly impact risk of PTB are unknown. However, identifying these mediating factors may illuminate options for policy remedies [18], such as those that remove barriers for at risk populations to foster educational opportunity. Furthermore, the Federal Neighborhood Stabilization Program, which was created to offer emergency assistance to stabilize neighborhoods with elevated rates of abandoned and foreclosed homes and to assist low income families, could be re-funded [76]. 

Future studies should examine what specific resources or ways of coping as well as the resiliency factors women who have higher education, and who reside in blighted neighborhoods, use that protects them from experiencing a PTB (for instance social capital). Future preterm birth reduction programs might target communities with high tax foreclosures, rather than or in addition to areas with high neighborhood poverty, and focus on women with lower educational attainment. A focus on leveraging community assets can help to reframe the disproportionate burden of PTB in African American communities as fixable, rather than intractable.

Our study has several strengths that distinguish it from the existing literature. First, we are the first to test the joint effect between educational attainment and neighborhood tax foreclosures and risk of PTB in urban African American women. Detroit, Michigan is an ideal place to study neighborhood effects on adverse birth outcomes given the striking racial residential segregation, urban blight, and economic disinvestment [77], combined with extreme racial disparities in PTB [78]. We leverage primary collected survey data (for individual-level social factors) and medical record data (for accurate gestational age ascertainment), and objective neighborhood tax foreclosure data, which improves upon and adds to the existing literature. We enrolled women in the immediate postpartum period, which means that our sample includes women with complete, interrupted or sporadic, and no prenatal care. In other words, the heterogeneity of risk for PTB in our study population was high, which likely increased the generalizability of our findings. Studies that recruit women from prenatal clinics may miss those who have incomplete or no prenatal care, a group that usually has the highest risk for PTB. 

Nevertheless, our study is not without limitations. First, all studies of neighborhood effects may be susceptible to neighborhood selection, which refers to the nonrandom sorting of individuals into neighborhoods [79,80,81]. To combat this, we adjusted our analysis by predictors of residential selection, including age, marital status, and income. The LIFE study was recruited from one suburban hospital, which could limit the generalizability of our findings, especially since our analysis was based on the subset of women who lived in Detroit. However, the overall LIFE sample had similar sociodemographic characteristics and birth outcomes as Non-Hispanic Black and African American women in the U.S., the State of Michigan, and Wayne County, MI [82]. In addition, the study recruitment hospital was chosen because of its wide catchment area, the heterogeneity of women receiving care (from 64 municipalities and 3 counties), and the large number of births per year [28]. Next, we examined the absolute number of neighborhood tax foreclosures, because we lacked appropriate data for the denominator to calculate true rates of neighborhood-level tax foreclosures. Future studies should quantify the relative impact of neighborhood tax foreclosures, their joint impact with other social exposures and potential mediating factors, and risk of adverse birth outcomes among urban residents. Given the cross-sectional nature of this research question, we are not able to make causal inferences, or to account for residential moves and exposures from other neighborhoods. Residual confounding by unmeasured or mis-measured factors and measurement error for our objective neighborhood measures cannot be ruled out. Future longitudinal research should quantify the impact of life-course neighborhood exposures on PTB risk among this high-risk group, and explicate the mediating pathways.

## 5. Conclusions

In summary, our findings suggest that the impact of neighborhood tax foreclosures on risk of PTB among African American women may depend on educational attainment. Women with >12 years of education may employ strategies and health behaviors that buffer them from the adverse effects, on PTB risk, of living in a neighborhood with increased blight and urban decline. Future work is needed to understand the mechanisms of these associations and to identify novel intervention targets, to decrease the persistently increased risk of PTB among African American women.

## Figures and Tables

**Table 1:** Subjective measures of the physical and social residential environment; Life Influences on Fetal Environments Study (2009–2011).

**Social Cohesion and Trust** (5-point Likert: Strongly agree, agree, neither agree nor disagree, disagree, strongly disagree)
1. I live in a close-knit neighborhood
2. People in my neighborhood are willing to help their neighbors
3. People in my neighborhood generally don’t get along with each other
4. People in my neighborhood do not share the same values
5. People in my neighborhood can be trusted
**Healthy Food Availability** (5-point Likert: Strongly agree, agree, neither agree nor disagree, disagree, strongly disagree)
1. A large selection of fresh fruits and vegetables is available in my neighborhood
2. A large selection of low fat products is available in my neighborhood
**Walkability** (5-point Likert: Strongly agree, agree, neither agree nor disagree, disagree, strongly disagree)
1. It is pleasant to walk in my neighborhood
2. The trees in my neighborhood provide enough shade
3. In my neighborhood it is easy to walk to places
4. I often see other people walking in my neighborhood
5. I often see other people exercise in my neighborhood
6. There are stores within walking distance of my home
**Safety** (5-point Likert: Strongly agree, agree, neither agree nor disagree, disagree, strongly disagree)
1. Many people in your neighborhood are afraid to go outside at night
2. There are areas of this neighborhood where everyone knows “trouble” is expected
3. You’re taking a big chance if you walk in this neighborhood alone after dark
4. I feel safe walking in my neighborhood
5. Violence is a problem in my neighborhood
6. I feel very safe from crime in my neighborhood
**Social Disorder** (3-point Likert: A big problem, somewhat of a problem, not a problem)
1. How much of a problem is litter, broken glass, or trash on the sidewalks and streets?
2. How much of a problem is graffiti on buildings and walls?
3. How much of a problem are vacant or deserted houses or storefronts?
4. How much of a problem is drinking in public?
5. How much of a problem is people selling or using drugs?
6. How much of a problem are groups of teenagers or adults hanging out in the neighborhood and causing trouble?
7. How much of a problem is noise in the neighborhood?
8. How much of a problem is yelling or fighting?

**Table 2:** Demographic characteristics of study participants and bivariate modified Poisson regression results; Life Influences on Fetal Environments Study (*n* = 686), 2009–2011.

	Term (*n* = 573) *n* (%)	PTB (*n* = 113) *n* (%)	RR	95% CI
Age				
18–19	47 (8.5)	9 (8.2)	1.17	0.58, 2.37
20–24	191 (33.3)	42 (37.2)	1.31	0.83, 2.08
25–29	151 (26.3)	24 (21.2)	*referent*	
30–34	94 (16.4)	21 (18.6)	1.33	0.78, 2.28
35+	90 (15.7)	17 (15.0)	1.16	0.65, 2.05
Marital status				
Single	443 (77.3)	82 (72.6)	*referent*	
Married	126 (22.0)	30 (26.6)	1.23	0.84, 1.80
Education (years)				
≤12	186 (32.5)	40 (35.4)	1.12	0.78, 1.58
>12	387 (67.5)	73 (64.6)	*referent*	
Income				
<$35,000	303 (52.9)	65 (57.5)	1.14	0.79, 1.64
≥$35,000	207 (36.1)	38 (33.6)	*referent*	
Time in neighborhood				
<24 months	254 (44.3)	50 (44.3)	1.00	0.71, 1.41
≥24 months	309 (53.9)	61 (54.0)	*referent*	
Perceived Social Cohesion				
Low	257 (47.2)	52 (48.6)	1.04	0.74, 1.48
High	287 (52.7)	55 (51.4)	*referent*	
Perceived Food availability				
Low	255 (45)	56 (50)	1.18	0.84, 1.66
High	312 (55)	56 (50)	*referent*	
Perceived Walkability				
Low	293 (42.5)	50 (44.6)	1.08	0.77, 1.51
High	324 (57.6)	62 (55.4)	*referent*	
Perceived Safety				
Low	244 (43.6)	53 (48.2)	1.17	0.83, 1.64
High	316 (56.4)	57 (51.8)	*referent*	
Perceived Disorder				
Low	261 (46.6)	47 (42.7)	*referent*	
High	299 (53.4)	63 (57.3	1.14	0.81, 1.61
Objective NDI				
Low	292 (51)	52 (46)	*referent*	
High	281 (49)	61 (54)	1.18	0.84, 1.65

PTB: Preterm birth; NDI: Neighborhood disadvantage index; RR: Relative risk; 95% CI: 95% Confidence Interval; percentages do not sum to 100 due to missing values (range: 0–11%).

**Table 3:** Modified Poisson regression results for associations between number of tax foreclosed properties at the block-group level and risk of preterm birth; overall and stratified by educational attainment; Life Influences on Fetal Environments Study (*n* = 686), 2009–2011.

Total Sample (*n* = 686)		≤12 Years Education (*n* = 226)		>12 Years Education (*n* = 460)	
Unadjusted RR (95% CI)	Adjusted RR (95% CI)	Unadjusted RR (95% CI)	Adjusted RR (95% CI)	Unadjusted RR (95% CI)	Adjusted RR (95% CI)
0.95 (0.77, 1.18)	0.93 (0.74, 1.16)	1.18 (0.84, 1.64)	1.31 (0.95, 1.82)	0.82 (0.63, 1.07)	0.74 (0.55, 0.98)

Adjusted for age, income, marital status, and time in current neighborhood; RR: Relative risk, CI: Confidence interval; neighborhood tax foreclosures X education *p* for interaction = 0.01.
